# An open-access dataset of global experimental yields in organic, diversified and conventional agricultural systems

**DOI:** 10.1016/j.dib.2026.112903

**Published:** 2026-05-30

**Authors:** Mahaman Sawadogo, Tamara Ben-Ari

**Affiliations:** UMR 0951 Innovation des systèmes agricoles et alimentaires, INRAE, CIRAD, SupAgro, Montpellier, France

**Keywords:** Yield stability, Agronomic experiments, Evidence synthesis, Long-term trials, Meta-analysis

## Abstract

The productive performance of organic and diversified agricultural systems remains the subject of intense debate. Despite a large body of empirical studies and several meta-analyses, an up-to-date, centralized dataset assembling medium- to long-term experimental evidence with annual yield time series is still lacking.

Here, we present an open-access global database of published field experiments comparing yields under organic or diversified management with conventional reference systems. The database compiles 102 peer-reviewed articles based on 73 experimental sites, representing 322 experimental units (i.e., unique yield comparisons). It provides standardized yield time series together with harmonized metadata describing crop species, geographic locations, experimental design, and key management practices (e.g., irrigation, fertilization, tillage, and diversification practices).

This dataset is designed to support reproducible syntheses of yield levels and yield variability across farming systems and crop types and to facilitate the integration of future long-term experimental results. The dataset is intended to support meta-analyses, model calibration, and scenario analyses of yield levels and yield stability across agricultural management systems.

Specifications Table

**Table:** 

Subject	Earth & Environmental Sciences
Specific subject area	*Crop production, farming systems, long-term field experiments*
Type of data	Table (CSV and Excel files); Raw
Data collection	*Merging of five published datasets and systematic literature review.*
Data source location	*23 countries across six continents*
Data accessibility	Repository name: Zenedo Data identification number: 10.5281/zenodo.18630006 Direct URL to data: https://doi.org/10.5281/zenodo.18630006 Instructions for accessing these data: The dataset is openly available on Zenodo under a CC-BY 4.0 license and can be accessed and downloaded directly via the DOI link without any restrictions [1].
Related research article	*None*

## 1 Value of the Data

• We provide a standardized and up-to-date database comparing organic, diversified and conventional agricultural yields in agricultural trials lasting at least four years.
• The database contains 102 published studies from 73 experimental sites corresponding to 322 experimental units (i.e., unique yield comparisons).
• The database enables comparisons of yield levels and yield variability across a wide range of cultivated species, accounting for agricultural practices (e.g., irrigation, fertilization).
• The structure of the database tables facilitates the integration of future experimental results.

## 2 Background

Alternative agricultural systems, including organic and diversified farming, provide numerous environmental benefits [2,3] and offer responses to pressing challenges such as biodiversity loss [4]. Their average yield per unit area is generally reported to be 8% to 25% lower than that of conventional agriculture, depending on crop type and location [[5], [6], [7]]. However, to date, most comparative assessments rely on short- to medium-term experiments. Moreover, whether yield stability differs systematically between systems remains an open question and requires evidence from medium- to long-term trials to robustly assess differences in yield variability.

Although numerous publications and several meta-analyses have compared the productive performance of conventional and organic, diversified, or no-till systems, an updated and centralized database of medium- to long-term experimental trials with annual yield information is still lacking. This data paper addresses this gap by providing a global database compiled from published experiments lasting 4 to 29 years and comparing yields under organic or diversified management (see definitions in Table 1 and principal characteritics in Table 2) with conventional reference systems. We also document the data selection and extraction procedures to ensure transparency, reproducibility, and facilitate future updates of the database.

## 3 Data Description

### 3.1 Experimental units

To compare yields under organic or diversified management with those under conventional management, we define an experimental unit (EU) as the smallest relevant scale of comparison. Each EU is characterized by: (i) the cultivated species; (ii) the study identifier and experimental site; (iii) the conventional reference treatment (e.g., high- or low-input, commercial, integrated, monoculture); (iv) the organic or diversified treatment (e.g., certified organic, biodynamic, intercropping, agroforestry); and (v) the treatment codes as reported in the original publications.

**Table 1:** Definitions of the three agricultural management systems.

Agricultural management systems	Characteristics
Organic	We define as organic any agricultural system following the IFOAM specifications (either certified or in transition) or biodynamic (i.e., purposely organic cropping systems)
Conventional	Conventional systems include any agricultural management explicitly reported as the conventional reference, including low-input systems when described as such in the original studies.
Diversified	Diversified systems are defined following [2] and include major forms of diversification at the global scale, such as crop rotation, intercropping, agroforestry, varietal mixtures and landscape heterogeneity.

**Table 2:** Classification of experimental units by country, continent and crop type. Percentages refer to the proportion of experimental units per category. Detailed information on species (Table S2) and countries (Table S1) is provided in the Supplementary Material.

Category	Class (%)
Country	Multiple countries
Continent (%)	North America (47.83); Europe (31.06); Asia (9.63); South America (5.28); Africa (4.04); Oceania (2.17)
Crop species	Multiple crop species (39 total)
Crop type (%)	Cereals (56.24); Oil crops (12.73); Vegetables (9.94); Tuber and root crops (4.66); Legumes (4.66); Beverage and spice crops (4.35); Forage crops (3.42); Fruits (2.80); Fiber crops (1.24)

This definition is more precise than that of the study itself, as it enables comparisons of organic or diversified and conventional yields for the same species, at the same site, and under comparable agronomic conditions, thereby limiting biases related to confounding factors. A single study may include several experimental units.

### 3.2 Data structure and content

**Fig. 1:**
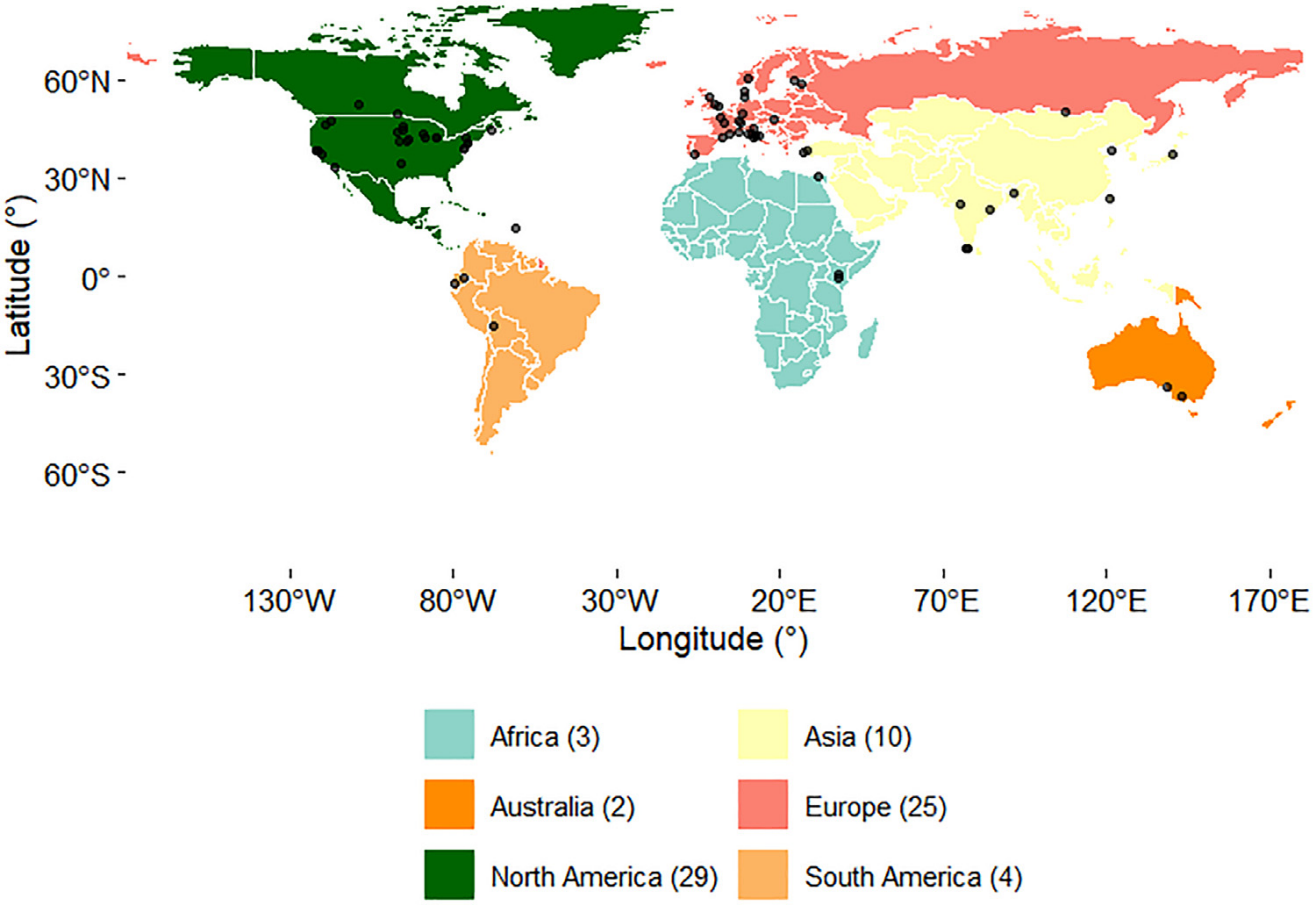
Geographic distribution of the experimental sites included in the database. Black circles indicate the location of each site based on reported latitude and longitude. Study sites are unevenly distributed across continents, with the highest concentration in the Northern Hemisphere, particularly in North America (29 sites), Europe (25 sites) and Asia (10 sites). The lowest number of sites is observed in Oceania (2 sites), followed by Africa (3 sites) and South America (4 sites).

The database is composed of 102 published articles (studies), corresponding to 322 unique comparisons (experimental units, EU). These data were collected from 73 experimental sites (some corresponding to multiple publications) located in 23 countries across the six continents (Fig. 1: Geographic distribution of the experimental sites included in the database. Black dots indicate the location of each site based on reported latitude and longitude. Study sites are unevenly distributed across continents, with the highest concentration in the Northern Hemisphere, particularly in North America (29 sites), Europe (25 sites) and Asia (10 sites). The lowest number of sites is observed in Oceania (2 sites), followed by Africa (3 sites) and South America (4 sites). In total, 39 cultivated species are represented, grouped into nine crop types, with four species accounting for 58.70% of all experimental units (see Table 3).

**Table 3:** Distribution of the most frequent crop species across experimental units.

Crop species	Frequency (%)
Wheat	19.25
Corn	19.88
Soybean	10.87
Barley	8.70
Total (4 species)	58.70

In addition to yield time series, the database includes harmonized metadata describing technical management practices, such as tillage, fertilization, and irrigation, as well as information on crop type, experimental design, and geographic context.

### 3.3 Overview of included studies

Five published meta-analyses were merged and complemented by an additional systematic literature review. The complete list of studies retained in the final database after screening by title, abstract, and full text, and the application of the inclusion criteria, is provided in Table S3 (Supplementary material). This table lists all articles included in the database, regardless of whether they originated from the merged meta-analyses or from the additional systematic search. The table contains 85 rows and three columns. Each row corresponds either to a single article or to a set of articles from the same long-term experimental site, and each column provides descriptive metadata, including the article title, authors, and year of publication.

**Fig. 2:**
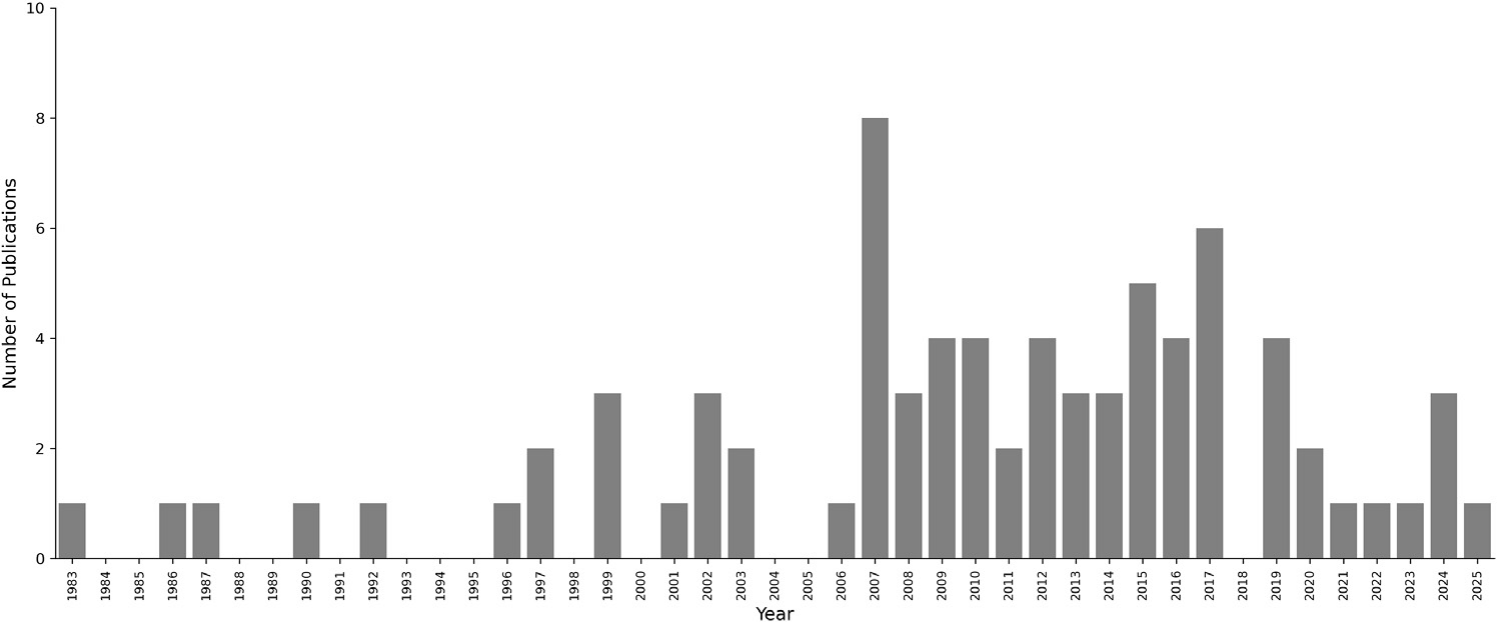
Temporal distribution of the publications included in the database. The histogram shows the number of articles published per year between 1983 and 2025. Note: Only dated publications are represented; undated publications are excluded from this representation.

The number of articles published each year is shown in Fig. 2.

### 3.4 Effect size

Effect size computation constitutes a core component of the database, as it enables standardized quantitative comparisons between agricultural management systems across heterogeneous experimental contexts. Three types of effect sizes are reported for each experimental unit: (i) the log mean yield ratio, (ii) the log variance ratio, and (iii) the log coefficient of variation ratio. For each ratio, an associated estimation of uncertainty is provided following the methodology proposed by [8].

A single study may contribute multiple effect sizes; for instance, when several years, crops, or treatments are reported within the same experimental site. For each experimental unit, the three aforementioned ratios were computed from the extracted annual yield data.

**Fig. 3:**
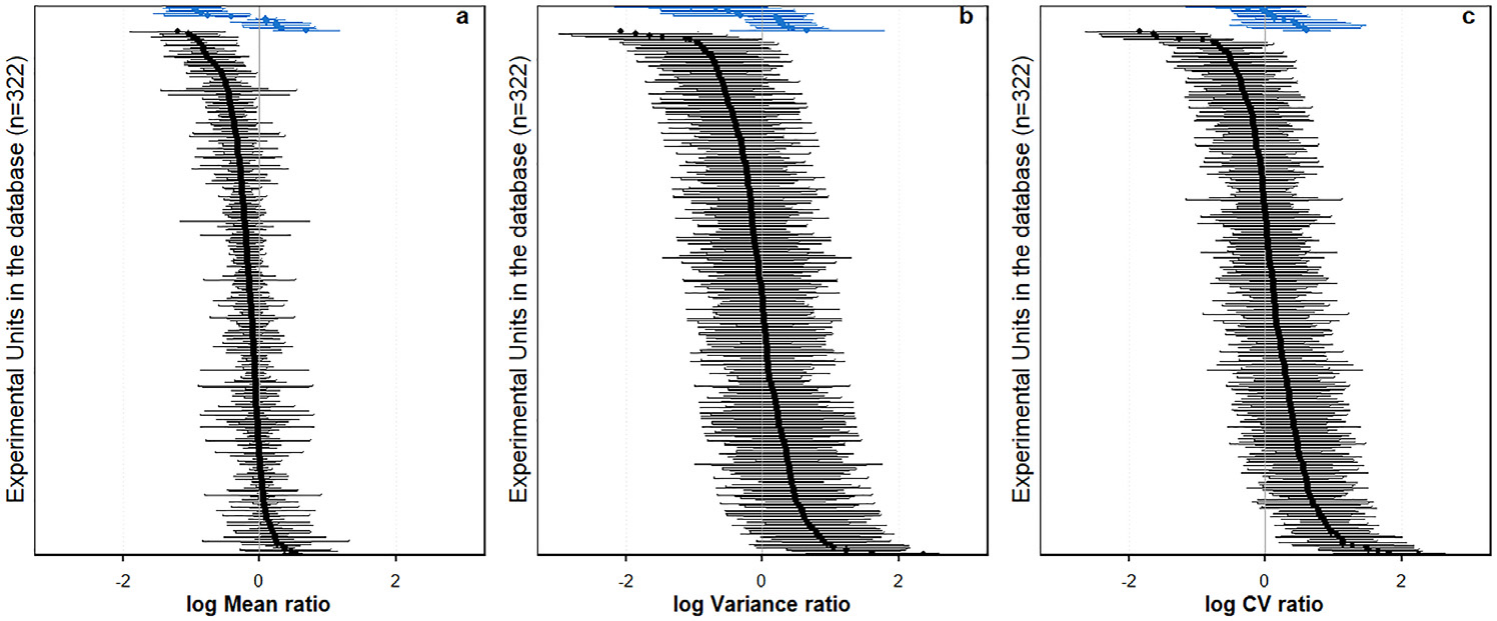
Effect sizes of agricultural management systems for each experimental unit. Panel (a) shows the log mean yield ratio (Eq. 1), panel (b) the log variance ratio (Eq. 2), and panel (c) the log coefficient of variation ratio (Eq. 3) for organic (black) or diversified (blue) systems. A log ratio equal to 0 indicates no difference between systems. For mean yield, values lower than 0 indicate higher productivity in conventional systems; for yield variance, values greater than 0 indicate stability in conventional systems. Points represent effect size estimates for each comparison, and horizontal bars indicate 95% confidence intervals.

**Fig. 4:**
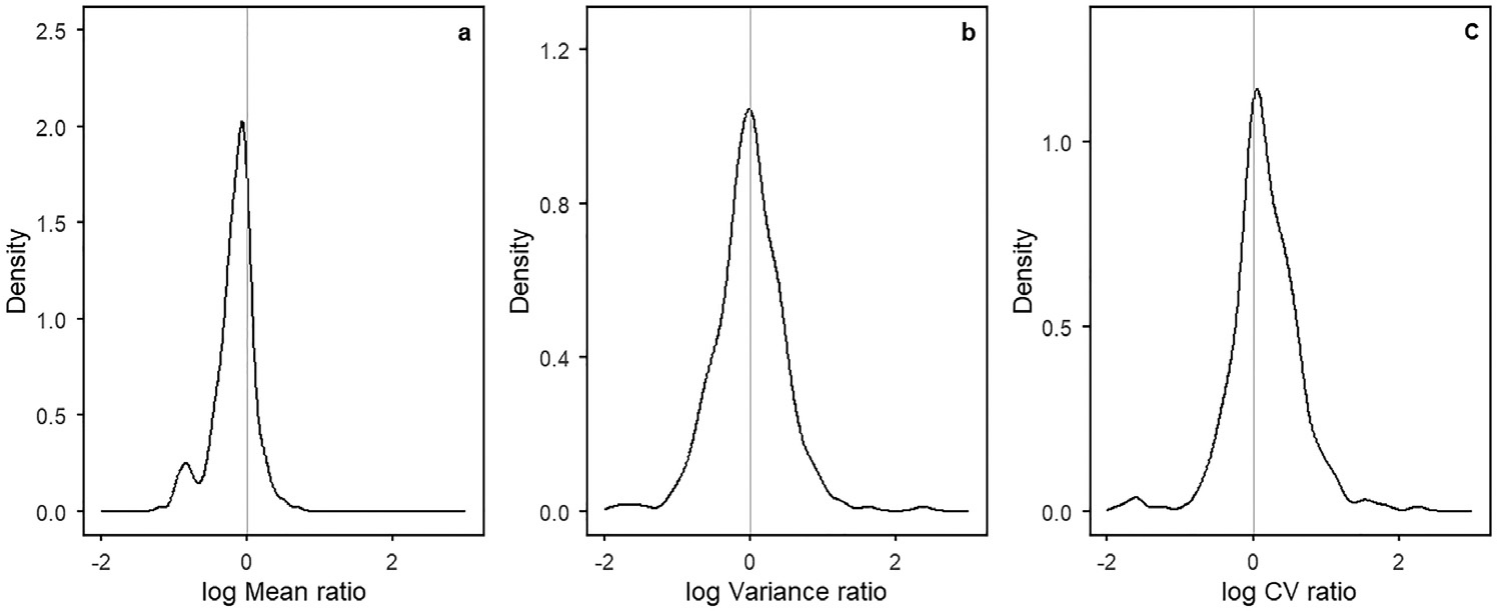
Probability distributions of the log mean yield ratio, log variance ratio and log coefficient of variation ratio across the entire dataset.

Forest plots illustrating the distribution of effect sizes and their associated 95% confidence intervals are provided in Fig. 3: Effect sizes of agricultural management systems for each experimental unit. Panel (a) shows the log mean yield ratio (Eq. 1), panel (b) the log variance ratio (Eq. 2), and panel (c) the log coefficient of variation ratio (Eq. 3) for organic (black) or diversified (blue) systems. A log ratio equal to 0 indicates no difference between systems. For mean yield, values lower than 0 indicate higher productivity in conventional systems; for yield variance, values greater than 0 indicate stability in conventional systems. Points represent effect size estimates for each comparison, and horizontal bars indicate 95% confidence intervals. The probability distributions of the three ratios across the entire dataset are shown in Fig. 4: Probability distributions of the log mean yield ratio, log variance ratio and log coefficient of variation ratio across the entire dataset.

Corrected effect sizes were calculated to compare alternative systems (diversified or organic) with conventional systems. For each experimental unit, the three effect sizes were calculated and confidence intervals (CI) as follows:

(1) $$log\;Mean\;Ratio=log\left(\frac{Mean_{org}}{Mean_{conv}}\right)$$

(2) $$log\;Variance\;Ratio=log\left(\frac{sd_{org}}{sd_{conv}}\right)+\;\frac{1}{2\left(N_{org}-1\right)}-\;\frac{1}{2\left(N_{conv\;}-1\right)}$$

(3) $$log\;CV\;Ratio=log\left(\frac{CV_{org}}{CV_{conv}}\right)+\;\frac{1}{2\left(N_{org}-1\right)}-\;\frac{1}{2\left(N_{conv}-1\right)}$$

$$CI_{1}=\log Mean\;Ratio\;\mp 1.96\;\times S_{\log Mean\;Ratio}$$

$$CI_{2}=\log Variance\;Ratio\;\mp 1.96\;\times S_{\log Variance\;Ratio}$$

$$CI_{3}=\log CV\;Ratio\;\mp 1.96\;\times S_{\log CV\;Ratio}$$

*With:*

$$S_{Mean\;Ratio}^{2}=\;\frac{sd_{org}^{2}}{N_{org}.Mean_{org}^{2}}+\;\frac{sd_{Conv}^{2}}{N_{conv}.Mean_{conv}^{2}}$$

$$S_{\log Variance\;Ratio}^{2}=\frac{1}{2\left(N_{\mathrm{org}}-1\right)}\;+\;\frac{1}{2\left(N_{\mathrm{conv}}-1\right)}$$

$$\begin{array}{ccc} S_{\log CV\;Ratio}^{2} & = & \;\left[\frac{sd_{org}^{2}}{N_{org}.Mean_{org}^{2}}+\frac{1}{2\left(N_{org}-1\right)\;}-2\rho \sqrt{\frac{sd_{org}^{2}}{N_{org}.Mean_{org}^{2}}\;.\frac{1}{2\left(N_{org}-1\right)}}\right]\\ & & +\left[\frac{sd_{Conv}^{2}}{N_{conv}.Mean_{conv}^{2}}+\frac{1}{2\left(N_{conv}-1\right)\;}\;-2\rho \sqrt{\frac{sd_{conv}^{2}}{N_{conv}.Mean_{conv}^{2}}\;.\frac{1}{2\left(N_{conv}-1\right)}}\right] \end{array}$$

*Where:*

*•* sd*: interannual standard deviation of yields for the system.*

*•* Mean*: interannual mean yield for the system.*

*•* N*: number of years of observation for the system.*

*•* ρ*: correlation between the log-mean and log-standard deviation for the system.*

*• S: sampling standard deviation.*

*•* $CI_{1\;}$*,* $CI_{2}$ *and* $CI_{3}$ *Denote respectively the confidence intervals of the Log Mean Ratio,*

*Log Variance Ratio, and Log CV Ratio.*

*For all effect sizes, positive values indicate higher values in alternative systems relative to conventional systems, whereas values close to zero indicate no difference between the two systems.*

## 4 Experimental Design, Materials and Methods

### 4.1 Article selection

The construction of the database followed a two-step procedure: (i) the merging of five existing databases [5,7,[9], [10], [11]] and (ii) an additional systematic literature search conducted in public bibliographic databases (Scopus and Web of Science) using the following search equation ((yield OR crop OR farming syst) AND ((organic OR diversif*) AND conventional) AND (agri* OR farm* OR product*) AND (irrigation OR rotation OR fert* OR tillage OR soil* OR multicrop* OR agroforest* OR intercrop*). The search is conducted without time limits, corresponding to publications from 1983 to 2025, and all redundant articles are removed in step (ii).

**Fig. 5:**
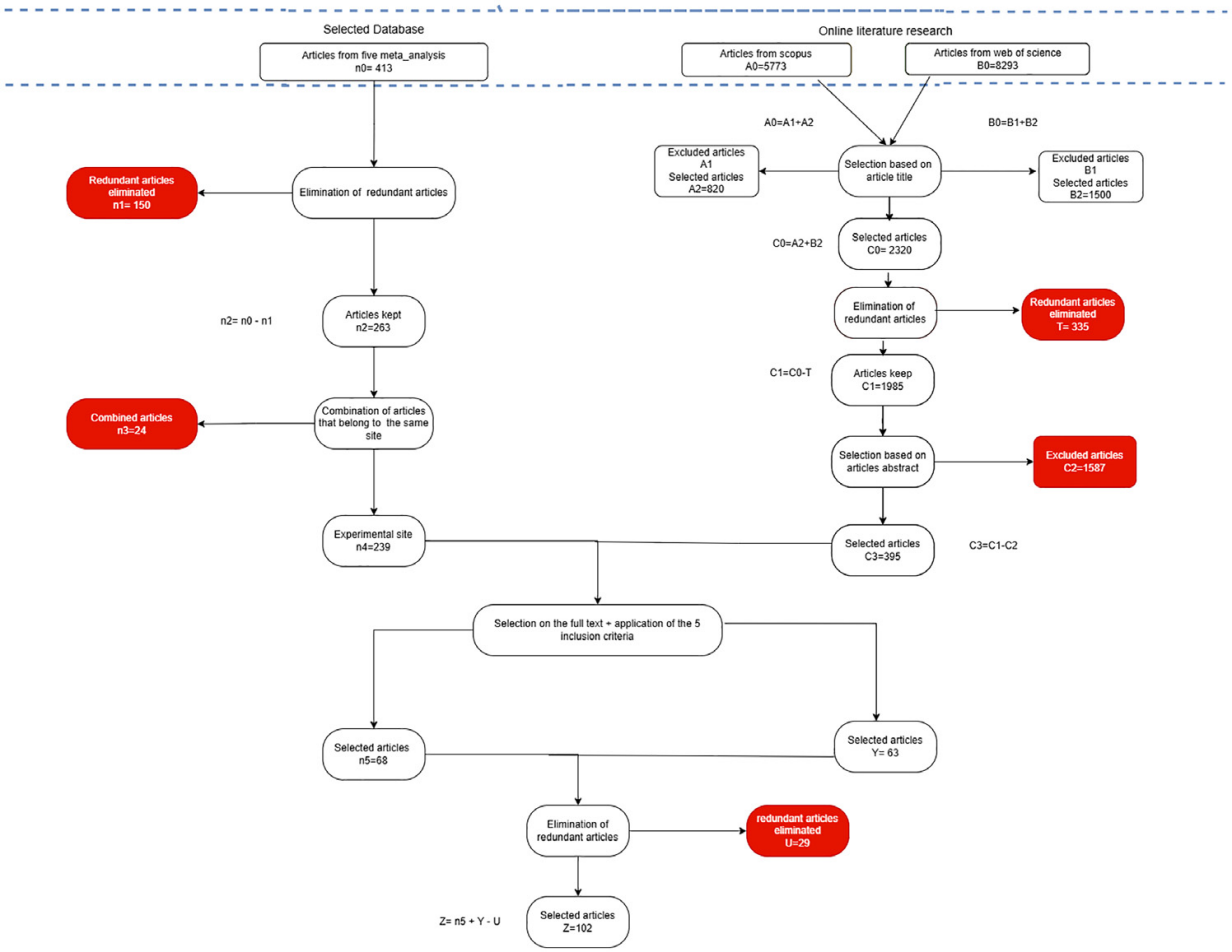
Workflow of the database construction process. The left-hand side shows the merging of five existing databases, followed by the same screening and selection steps. The right-hand side depicts the selection of articles from Web of Science and Scopus, including screening by title, abstract and full text, and application of the inclusion criteria.

In a first step, the data from the five most recent meta-analyses comparing organic and conventional yields and published between 2012 and 2017 were merged, resulting in 413 articles, with each database containing between 15 and 150 studies. Results already included in one of the other selected meta-analyses, as well as studies lacking a conventional control treatment at the plot level, were excluded, resulting in 263 articles. Then, articles presenting results from the same experimental site were combined, resulting in 239 articles. Subsequently, the following inclusion criteria are applied: (i) yield comparisons between organic and conventional systems for the same crop species, (ii) comparisons conducted at the same experimental site, (iii) within the same year, and (iv) over a minimum duration of four years (consecutive or non-consecutive). This first step results in the selection of 68 articles, see Fig. 5: Workflow of the database construction process. The left-hand side shows the merging of five existing databases, followed by the same screening and selection steps. The right-hand side depicts the selection of articles from Web of Science and Scopus, including screening by title, abstract and full text, and application of the inclusion criteria.

The second step consists of a systematic literature search in Scopus and Web of Science using Boolean search equations specifically designed to capture studies comparing organic to conventional farming systems and diversified to conventional systems. The search was restricted to peer-reviewed articles published in English, French, German, Italian, and Spanish. This extraction provided 5773 and 8293 articles in Scopus and Web of Science, respectively.

The screening process involved successive filtering steps. First, records were screened based on titles, resulting in the selection of 1,985 articles after the removal of duplicates. A second screening was then performed based on abstracts, yielding 395 articles after further deduplication. The same inclusion criteria as those applied to the merged databases were subsequently used to assess full-text eligibility, leading to the identification of 63 additional articles meeting all inclusion criteria. Among these, 29 were duplicates of studies already retained at the first step and were therefore excluded, leading to the selection of 63 articles. Overall, this two-step selection procedure resulted in a final dataset of 102 independent articles included in the database. A flow diagram summarizing the merging and screening procedure is provided in Fig. 5.

### 4.2 Data extraction and quality assessment

Yield data and associated metadata (article title, authors, year of publication, experimental site, crop species, and management practices) were extracted from the selected 63 published articles not available in existing meta-analyses using information reported in Fig.s, tables, and the main text. For 21 articles in which annual yield values were only available in graphical form, data were digitized using WebPlotDigitizer. In addition, corresponding authors were contacted for 43 studies when yield information was insufficiently detailed (e.g., only multi-year averages were reported) to retrieve annual yield data.

Data quality control was implemented at several stages of the database construction process, including during article screening and data extraction. An external reviewer independently verified the selection procedure. Each included article was examined multiple times to ensure the accurate extraction of quantitative and qualitative information. In cases of uncertainty or ambiguity, a second reviewer was consulted to provide an independent assessment.

To further assess data reliability, sensitivity analyses and checks for potential selection bias were conducted using standard statistical approaches. All extracted data were systematically checked for internal consistency using R software. Finally, extreme values were examined by comparison with the original sources to confirm their validity.

## Limitations

Meta-analyses and data compilations are subject to recurring limitations, mainly related to (i) possible biases in article selection and (ii) difficulties or errors in data extraction. In the present study, the second phase of the systematic search revealed that two articles initially selected based on their abstracts could not be accessed, even through alternative bibliographic sources. In addition, several studies reported only aggregated yield information (e.g., mean yields over multiple trial years), which required contacting the corresponding authors to obtain annual yield data. Of the 43 authors contacted, 14 provided the requested information, 29 did not respond, and contact could not be established for six authors due to invalid or outdated email addresses.

**Fig. 6:**
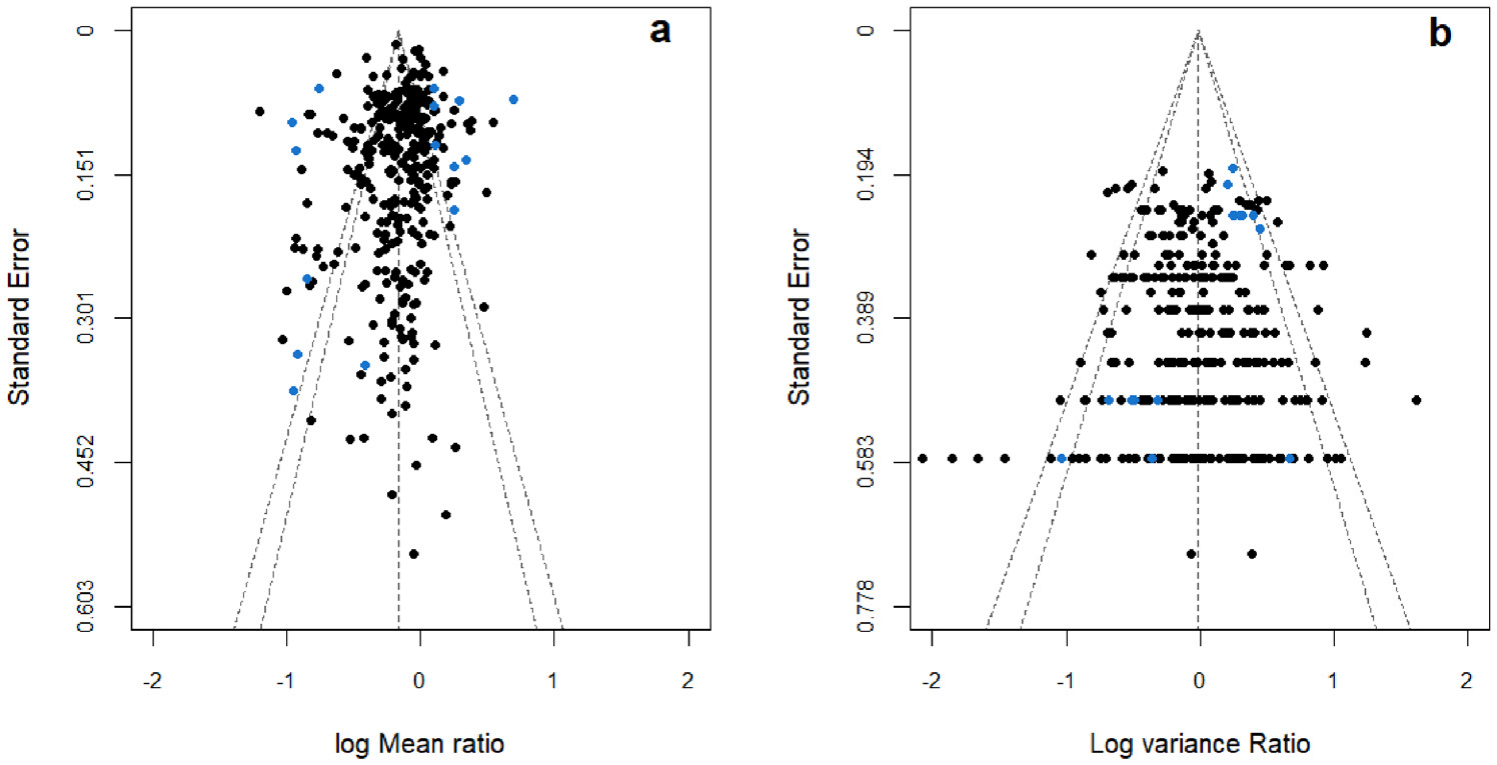
Funnel plots of effect sizes based on (a) the log Mean yield ratio and (b) the log Variance ratio are provided to assess potential small-study effects and publication bias, for comparisons between organic vs. conventional (black points) and diversified vs. conventional (blue points). No strong asymmetry is visually apparent in these plots.

Funnel plots suggest that a bias towards negative log mean yield ratios (i.e., higher yields in conventional systems) cannot be excluded. Whether this reflects an over-representation of studies conducted in conventional systems or other factors remains to be determined as the database expands. This pattern appears less pronounced for comparisons involving diversified systems (Fig. 6: Funnel plots of effect sizes based on (a) the log Mean yield ratio and (b) the log Variance ratio are provided to assess potential small-study effects and publication bias, for comparisons between organic vs. conventional (black points) and diversified vs. conventional (blue points). No strong asymmetry is visually apparent in these plots.

These limitations highlight the importance of continued data collection and updating the database as new long-term experimental results become available.

## Ethics Statement

The authors have read and follow the ethical requirements for publication in Data in Brief. The current work does not involve human subjects, animal experiments, or any data collected from social media platforms.

## CRediT Author Statement

**Tamara Ben-Ari:** Conceptualization, Methodology, Investigation, Visualization, Supervision, Writing -original draft, Writing - review & editing. **Mahaman Sawadogo:** Investigation, Visualization, Writing - original draft, Writing - review & editing.

## Data Availability

ZenodooGlobal database of long-term experimental yields in organic, diversified and conventional agricultural systems (Original data)

## Appendix Supplementary materials

Image, application 1

Image, application 2

## References

[1] Sawadgo M., Ben-Ari T. (2026). Global database of long-term experimental yields in organic, diversified and conventional agricultural systems. Zonedo.

[2] Beillouin D., Ben-Ari T., Makowski D. (2019). Evidence map of crop diversification strategies at the global scale. Env. Res. Lett..

[3] Beillouin D., Ben-Ari T., Malézieux E., Seufert V., Makowski D. (2021). Positive but variable effects of crop diversification on biodiversity and ecosystem services. Glob. Change Biol..

[4] Tscharntke T., Grass I., Wanger T.C., Westphal C., Batáry P. (2021). Beyond organic farming–harnessing biodiversity-friendly landscapes. Trends. Ecol. Evol..

[5] Hossard L. (2016). A meta-analysis of maize and wheat yields in low-input vs. Conventional and organic systems. Agron. J..

[6] Knapp S., van der Heijden M.G. (2018). A global meta-analysis of yield stability in organic and conservation agriculture. Nat. Commun..

[7] Seufert V., Ramankutty N., Foley J.A. (2012). Comparing the yields of organic and conventional agriculture. Nature.

[8] Nakagawa S. (2015). Meta-analysis of variation: ecological and evolutionary applications and beyond. Methods Ecol. Evol..

[9] Lesur-Dumoulin C., Malézieux E., Ben-Ari T., Langlais C., Makowski D. (2017). Lower average yields but similar yield variability in organic versus conventional horticulture. A meta-analysis. Agron. Sustain. Dev..

[10] De Ponti T., Rijk B., Van Ittersum M.K. (2012). The crop yield gap between organic and conventional agriculture. Agric. Syst..

[11] Ponisio L.C., M’Gonigle L.K., Mace K.C., Palomino J., De Valpine P., Kremen C. (2015). Diversification practices reduce organic to conventional yield gap. Proc. R. Soc. B.

