# Supplementary material for: An open-access dataset of global experimental yields in organic, diversified and conventional agricultural systems
Source: Data Brief. 2026 May 30;67:112903. doi: 10.1016/j.dib.2026.112903 (PMC13264129; doi:10.1016/j.dib.2026.112903)
Supplement: Supplementary file 1 [file mmc1.pdf]

An open-access global database investigating experimental yields in organic, diversified and conventional agricultural systems

Mahaman Sawadogo[1] and Tamara Ben Ari*[1]

[1] *UMR 0951 Innovation des systèmes agricoles et alimentaires, INRAE, CIRAD, SUpAgro, Montpellier, France*

Abstract

Organic and diversified agricultural systems are widely promoted for their potential benefits for biodiversity and environmental sustainability; yet, their comparative stability remains debated. Although numerous empirical studies and meta-analyses have compared yields across farming systems, an up-to-date and centralized dataset compiling medium- to long-term experimental evidence with annual yield data is still lacking.

Here, we present an open-access global database of published field experiments comparing yields under organic or diversified management with conventional reference systems. The database compiles 102 peer-reviewed articles based on 73 experimental sites, representing 322 experimental units (*i.e.*, unique yield comparisons). It provides standardized yield time series together with harmonized metadata describing crop species, geographic locations, experimental design, and key management practices (*e.g.*, irrigation, fertilization, tillage, and diversification practices). This dataset is designed to support reproducible syntheses of yield levels and yield variability across farming systems and crop types and to facilitate the integration of future long-term experimental results.

Keywords: meta-analysis, yield stability, agronomic trials.

---

*Corresponding author: `tamara.ben-ari@inrae.fr`

Supplementary Material

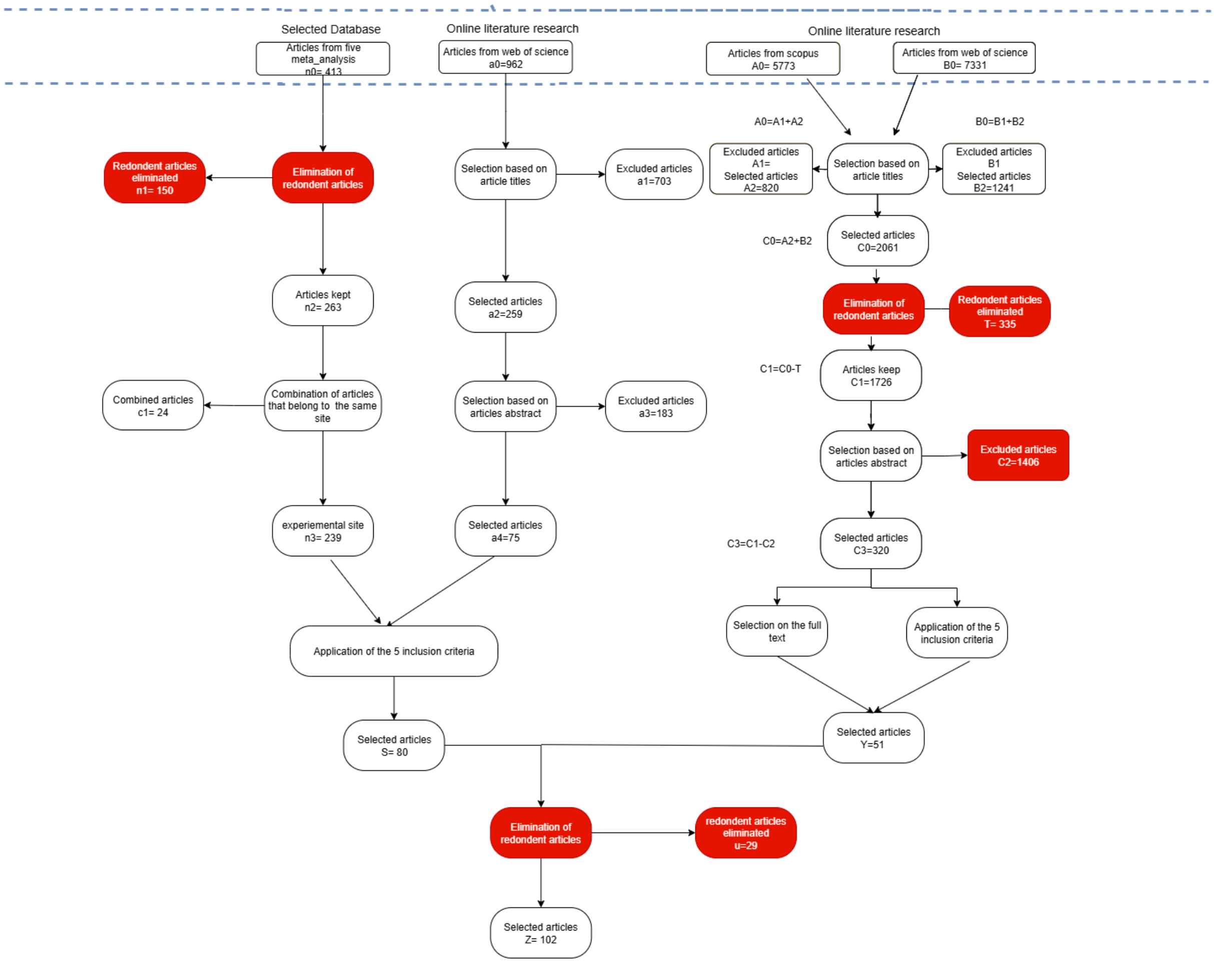

Figure S1: The Sankey diagram shows the details of the two phases of database construction. The first phase on the left, and the update phase on the right.

Table S1: Countries included in the database

**Category**	**Class**
**Country**	USA
	France
	Denmark
	Norway
	Italy
	Switzerland
	India
	Ecuador
	Germany
	Japan
	United Kingdom
	Slovakia
	Martinique
	Kenya
	Turkey
	Taiwan
	Spain
	Bolivia
	Canada
	Australia
	Espagne
	Egypt
	Estonia
	Finland

Table S2: Crop species included in the database

Category	Class
**Crop species**	Corn
	Wheat
	Soybean
	Rice
	Tomato
	Barley
	Oat
	Cauliflower
	Apple
	Elephant foot yam
	Alfalfa
	Banana
	Beetroot
	Carrot
	Potato
	Rye
	Pigeon pea
	Bean
	Safflower
	Tahiti lime
	Babycorn
	Grapes
	Greater yam
	Lesser yam
	White yam
	Taro
	Silage corn
	Temporary grassland
	Rapeseed
	Cotton
	Lettuce
	Cocoa
	Flaxseed
	Cabbage
	French bean
	Faba bean
	Coffea
	Muskmelon
	Cucumber
	Pepper

Table S3: Table of studies included in our systematic review

Author(s)	Year	Title
Antichi et al.	2025	Long-term evaluation of organic management of durum wheat in central Italy
Bautze et al.	2024	Closing the crop yield gap between organic and conventional farming systems in Kenya: Long-term trial research indicates agronomic viability
Sabahy et al.	2024	Enhancing Water Use Efficiency and Carbon Profitability Through the Long-Term Impact of sustainable Farming systems
Jaramilo et al.	2024	Nutrient contribution and carbon sequestration of an agroforestry system of Coffea canephora cultivated by conventional and organic management in the Ecuadoran Amazon
Notaris et al.	2023	Faba bean productivity, yield stability and N2-fixation in long-term organic and conventional crop rotations
Verdi et al.	2022	Comparison between organic and conventional farming systems using Life Cycle Assessment (LCA): A case study with an ancient wheat variety
Logsdon et al.	2021	Organic agriculture effect on water use, tile flow, and crop yield
Bender et al.	2020	Organic carrot (Daucus carota L.) production has an advantage over conventional in quantity as well as in quality
Fan et al.	2020	Rotational benefit of pulse crop with no till increase over time in a semiarid climate
Armstrong et al.	2019	Effects of long term rotation and tillage practice on grain yield and protein of wheat and soil fertility on a vertosol in a medium-rainfall temperate environment
Armstrong et al.	2019	Effects of long term rotation and tillage practice on grain yield and protein of wheat and soil fertility on a vertosol in a medium-rainfall temperate
Li et al.	2019	Yields and resilience outcomes of organic, cover crop, and conventional practices in a Mediterranean climate
Niether et al.	2019	Below- and aboveground production in cocoa monocultures and agroforestry systems
Tosti et al.	2019	Processing Tomato–Durum Wheat Rotation under Integrated, Organic and Mulch-Based No-Tillage Organic Systems Yield, N Balance and N Loss
WICST	2017	Yearly Crop Yields on the WI Integrated Cropping Systems Trial

Continued on next page

Table S3: Table of studies included in our systematic review (continued).

Author(s)	Year	Title
Musyoka et al.	2017	Effect of organic and conventional farming systems on nitrogen use efficiency of potato, maize and vegetables in the Central highlands of Kenya
Das et al.	2017	Impact of seven years of organic farming on soil and produce quality and crop yields in eastern Himalayas, India
Suja et al.	2017	Yield, quality and soil health under organic vs conventional farming in taro
Lychuk et al.	2017	Effects of crop inputs, diversity, environment, and terrain on yield in an 18-yr study in the semi-arid Canadian Prairies
Dal Ferro et al.	2017	Crop yield and energy use in organic and conventional farming: A case study in north-east Italy
Bertrand et al.	2016	Design of sustainable and innovative cropping systems for arable crops. The case of the long-term experiment 'La Cage' in Versailles, France.
Adamtey et al.	2016	Productivity, profitability and partial nutrient balance in maize-based conventional and organic farming systems in Kenya
Armengot et al.	2016	Cacao agroforestry systems have higher return on labor compared to full-sun monocultures
Okur et al.	2016	A comparison of soil quality and yield parameters under organic and conventional vineyard systems in Mediterranean conditions (West Turkey)
Mayer et al.	2015	Productivity, quality and sustainability of winter wheat under long-term conventional and organic management in Switzerland
Sacco et al.	2015	Six-year transition from conventional to organic farming: effects on crop production and soil quality
Campiglia et al.	2015	The long-term effects of conventional and organic cropping systems, tillage managements and weather conditions on yield and grain quality of durum wheat (Triticum durum Desf.) in the Mediterranean environment of Central Italy
Farneselli et al.	2015	Yield and apparent dry matter and nitrogen balances for muskmelon in a long-term comparison between an organic and a conventional low input cropping system
Duman et al.	2015	A long-term trial to determine variations in the yield and quality of a processing type pepper (Capsicum annuum L. cv. Yalova yağlık-28) in organic and conventional farming systems

Continued on next page

Table S3: Table of studies included in our systematic review (continued).

**Author(s)**	**Year**	**Title**
Suja & Sreekumar	2014	Implications of organic management on yield, tuber quality and soil health in yams in the humid tropics
Chong-Ho Wang	2014	Farming Methods Effects On The Soil Fertility And Crop Production Under A Rice-Vegetables Cropping Sequences
Montemurro et al.	2014	Cropping systems, tillage and fertilization strategies for durum wheat performance and soil properties
Palmer et al.	2013	The influence of organic and conventional fertilisation and crop protection practices, preceding crop, harvest year and weather conditions on yield and quality of potato (Solanum tuberosum) in a long-term management trial
Bilsborrow et al.	2013	The effect of organic and conventional management on the yield and quality of wheat grown in a long-term field trial
Forster et al.	2013	Yield and economic performance of organic and conventional cotton-based farming systems–results from a field trial in India
Korsaeth	2012	N, P, and K Budgets and changes in selected topsoil nutrients over 10 years in a long-term experiment with conventional and organic crop rotations
Suja et al.	2012	Higher yield, profit and soil quality from organic farming of elephant foot yam
Behera et al.	2012	Effects of Fertilizers on Yield, Sustainability, and Soil Fertility under Rainfed Pigeon pea, Rice System in Subhumid Oxisol Soils
Hokazono et al.	2012	Variability in environmental impacts during conversion from conventional to organic farming: a comparison among three rice production systems in Japan
Delmotte et al.	2011	On farm assessment of rice yield variability and productivity gaps between organic and conventional cropping systems
Wheeler & Crisp	2011	Going organic in viticulture: a case-study comparison in Clare Valley, South Australia
Doltra et al.	2010	Cereal yield and quality as affected by nitrogen availability in organic and conventional arable crop rotations: A combined modeling and experimental approach
Peck et al.	2010	Integrated and organic fruit production systems for 'Liberty' apple in the Northeast United States: a systems-based evaluation

Continued on next page

Table S3: Table of studies included in our systematic review (continued).

Author(s)	Year	Title
Russo et al.	2010	Frequency of manure application in organic versus annual application of synthetic fertilizer in conventional vegetable production
Zihlmann et al.	2010	Integrierter und biologischer Anbau im Vergleich
Cavigelli et al.	2009	Long-term economic performance of organic and conventional field crops in the mid-Atlantic region
Ryan et al.	2009	Weed–crop competition relationships differ between organic and conventional cropping systems
Pardo et al.	2009	Economic Evaluation of Cereal Cropping systems Under Semiarid Conditions: Minimum input, organic and conventional
Welsh et al.	2009	High yielding organic crop management decreases plant-available but not recalcitrant soil phosphorus
Lo Scalzo et al.	2008	Organic vs conventional Field Trials: the Effect on Cauliflower
Lavigne et al.	2008	Culture biologique de la lime de Tahiti à la Martinique
Wang et al.	2008	Summer cover crop and in-season management system affect growth and yield of lettuce and cantaloupe
Archer et al.	2007	Crop Productivity and Economics during the Transition to Alternative Cropping Systems
Teasdale et al.	2007	Potential long-term benefits of no-tillage and organic cropping systems for grain production and soil improvement
Jimenez et al.	2007	Organic banana production in Ecuador: Its implications on black Sigatoka development and plant-soil nutritional status
Herencia et al.	2007	Comparison between organic and mineral fertilization for soil fertility levels, crop macronutrient concentrations, and yield
Swezey et al.	2007	Six-year comparison between organic, IPM and conventional cotton production systems in the Northern San Joaquin Valley, California
WICST	2007	WICST Annual Crop Yields
Alyson et al.	2007	Ten-year comparison of the influence of organic and conventional crop management practices on the content of flavonoids in tomatoes
Braman	2006	Soil Health After 19 Years Under Organic and Conventional Agriculture and Restored Prairie Grassland

Continued on next page

Table S3: Table of studies included in our systematic review (continued).

**Author(s)**	**Year**	**Title**
Glenlea	2005	Glenlea Long-Term Crop Rotation: 2005 Yield Results
Porter et al.	2003	Organic and other management strategies with two- and four-year crop rotations in Minnesota
Letter et al.	2003	The performance of organic and conventional cropping systems in an extreme climate year
Maeder et al.	2002	Soil fertility and biodiversity in organic farming
Paudel et al.	2002	Comparison of soil N availability and leaching potential, crop yields and weeds in organic, low-input and conventional farming systems in northern California
Reganold et al.	2001	Sustainability of three apple production systems
Clark et al.	1999	Crop-yield and economic comparisons of organic, low-input, and conventional farming systems in California's Sacramento valley
Lacko-Bartsova et al.	1999	Effect of ecological and integrated arable farming systems on crop productivity and soil fertility
Raupp	1999	Entwicklung des Kornertrages und der Ertragskomponenten von Winterroggen in einem Langzeit-Duengungsversuch
Delate et al.	1998–2015	Comparison of Organic and Conventional Corn, Soybean, Alfalfa, Oats, And Rye Crops at the Neely-Kinyon Long-Term Agroecological Research (LTAR)
Dobbs & Smolik	1997	Productivity and profitability of conventional and alternative farming systems: A long-term on-farm paired comparison
Cormack et al.	1997	Organic arable systems at ADAS Terrington
Raupp	1996	Quality of plant products grown with manure fertilization
Besson et al.	1992	DOK-Versuch: vergleichende Langzeituntersuchungen in den drei Anbausystemen biologisch-dynamisch, organisch-biologisch und konventionell. II. Ertrag der Kulturen: Gerste, 1. und 2. Fruchtfolgeperiode
Reganold et al.	1987	Long-term effects of organic and conventional farming on soil erosion
Helmers et al.	1986	An economic analysis of Alternative cropping systems for east-central Nebraska
Eggert F.P. et al.	1983	Effect of soil management practices on yield and foliar nutrient concentration of dry bean, carrots, and tomatoes.

Continued on next page

Table S3: Table of studies included in our systematic review (continued).

**Author(s)**	**Year**	**Title**
Ward J.P. et al.	1952–1965	The Haughley experiment.
LTRAS/Century experiment, UC Davis	undated	NA
KBS	undated	KBS Main Cropping System Experiment (MCSE)
KBS	undated	KBS Living Field Lab (LFL)
KBS	undated	KBS Field-scale experiment
Goldstein et al.	undated	Comparisons of conventional, organic, and biodynamic methods
